# Recollections and Observations of a Country Practice, with Cases1Abstract of a paper read before the Wyoming County Medical Association, at Castile, October 29, 1895.

**Published:** 1896-01

**Authors:** George M. Palmer

**Affiliations:** Warsaw, N. Y.


### RECOLLECTIONS AND OBSERVATIONS OF A COUNTRY PRACTICE, WITH CASES.[1]

BY GEORGE M. PALMER, M. D., Warsaw, N. Y.

I SUPPOSE, because of rural associations and lack of polish incident to newer and more enlightened opportunities, under which the later generations have been trained, it is natural to look upon old-time workers as professional back numbers.

I have regretted somewhat my promise to occupy your time, because of the painful conditions surrounding me, necessarily breaking in upon the train of thought, tending, naturally, to make it disconnected and incomplete but retrospective, simply recalling my early experiences and observations, making no claims beyond what a moderate degree of professional wisdom, as taught half a century ago, and a fair degree of practical common sense, would justify.

The practical points that I hope to make are: first, the importance of being the possessor of practical common sense and a good stock of versatile mental resources in emergencies, and the ability to think; second, the importance of cleanliness in person and in work; third, to call attention to the wonderful advances that are being made in the science of medicine and surgery, emphasising

1. Abstract of a paper read before the Wyoming County Medical Association, at Castile, October 29, 1895.

the thought that the later graduates in medicine are not to be excused if they are not better equipped for their work than were their long-ago predecessors.

The emergencies that beset the lives of medical men have proved that they must be thinkers, not only with mental apparatus intended to fulfil that function, but with all of the accessories that bind together and obey the mandates of the brain. All of the organs of special sense must be trained, practical and alert, and each heart-beat feel the impulse born of a great love for, and pride in, the work he has to do and a care for its results, that they reflect a light that shall illuminate his life with gratitude for what he has accomplished, and a hope for and faith in a successful ending of the work that lies before him.

Some of us here present can remember the teaching that the point of the finest cambric needle piercing the peritoneum was sure to induce serious or fatal inflammation, and that opening into a diseased joint, allowing the admission of air, was sure to bring serious, if not fatal, results.

Something must happen to set the profession thinking upon the subject, and Providence gave them the case of the insane woman, who plunged a large pair of tailors' shears into her abdomen, allowing several knuckles of intestine to protrude from the wounds. Thorough irrigation with hot salt water, returning the protruding intestines, closing the wounds and opium narcosis, permitted the woman to recover with reason restored. Another, the case in point is that of one of my own patients, who with a razor mutilated himself and made a large opening into his abdomen, through which his intestines protruded. The wounds and protrusions were thoroughly cleansed with hot salt water, some bleeding vessels ligated, the abdominal contents replaced, sutures introduced, and the hot brine treatment, with compresses and bandages and narcotics, served to effect a cure. Such incidents caused the profession to wonder and to speculate.

I remember once seeing some professional gentlemen gather up their belongings and, with the motion of washing their hands of such a proceeding, leave me alone with the patient, because I insisted that a large free incision should be made into a knee-joint that all admitted was surrounded by and filled with pus. They remembered about valvular openings into joints. Nevertheless, a four-inch opening was made, air admitted and pus discharged, and the best then known aseptic treatment, thorough continuous irrigation with boiled water, was resorted to. This patient will, today, walk or run a foot race with any man of his age.

How few the years since laparatomy, at the hands of two or three distinguished surgeons under the name of ovariotomy, was regarded as an almost superhuman exploit, and a history of 30 to 50 per cent. of recoveries in selected cases was considered something marvelous; while now at the hands of expert operators strings of patients, from the highest grade of humanity down to the lowest denizens of the slums, are walking out of our hospitals after those operations, and the fatal cases are hardly worth recording, so insignificant is the number, and country practitioners in almost every town and hamlet, where within my memory it was thought necessary that a patient must seek a hospital to have a finger amputated, are turning out successful laparatomies. As I go on I shall cite a few cases under the old methods, which will fulfil my purpose and suffice for comparisons:

Case I.—A. G., aged 50 years, was dragged by a runaway team, striking with great violence against a stationary obstacle. The blow was received on the lower border of the left orbit and malar bone. The whole of the bony structures were driven downward until the superior maxilla and palate bones were almost on edge, the lower maxilla fractured and forced into the position seen in its complete dislocation. The soft parts were bruised and lacerated and the patient unconscious. The attending physician had decided that there was not much to do. I was called in consultation a few hours later. Then the hemorrhage had subsided and the blood stains partially removed, giving the patient a less formidable appearance. Fortunately, the accident occurred in winter, and snow, instead of gravel and dirt, was ground into the injured parts. All ordinary manual efforts failed to move the bones, and so a leverage was devised, and finally, after and during thorough irrigation with water, the broken parts were loosened and placed in position. The nasal bones were crushed and the malar prominence had disappeared. The fenestrated ends of large, flexible catheters were pushed well up into the nasal passages, loosened teeth removed, softened paraffine fitted between and around the remaining molars, retention bandages applied and continuous irrigation directed with a solution of potassium chlorate. The subsequent dressings were almost wholly water. This patient made an excellent recovery. There was very little pus and that was mainly in the nasal passages, probably due, in great measure, to the presence of the catheters.

Case II.—D. J., aged about 25 years, was kicked squarely in his face by a powerful young horse. I was called by Dr. S. C. Smith, of Castile, to see the case with him. Examination revealed that the bones of the face and upper maxilla were crushed and comminuted, no parts below the orbits presenting any appearance of the human face. Considerable time was spent in irrigation. The face and oral cavity could be molded and shaped readily by gentle manipulation, only to drop back again to the original shapeless condition. A little knowledge of dentistry came to our aid, and an impression was made with softened wax and molded into form, and from this a vulcanised rubber obturator plate was made and fitted as a splint. The injured parts were kept in place, and this patient got up with very slight deformity or impairment of function. In this case, too, water dressings were relied upon.

CASE III.—A. Mc., aged 19, was attacked with violent pains in right hypogastrium whilst following the plow. He was carried to the house, placed in bed and the ordinary domestic remedies resorted to. No medical aid, however, was sought until some weeks had passed, when his father came to my office with the statement that he thought his son was fatally ill. I found the patient emaciated, haggard and almost idiotic ; his right thigh was strongly flexed upon the body, and a large fluctuating tumor in the right epigastrium, which evidently contained pus quite near the surface. I made a pretty free incision, which permitted the escape of a large quantity of pus having an intensely fecal odor. The patient fainted, but under the use of restoratives he soon revived, when the opening was enlarged. The abscess cavity was flushed with hot salt water, packed pretty firmly with finely-picked oakum, and constant irrigation with a solution of phenol and potassium chlorate at 100° F. for the next twenty-four hours, by means of a siphon. At my next visit the patient was in a condition that deterred me from disturbing the packing and I left him with the siphon in operation, which was continued until the fourth day, when the packing was removed and the cavity, somewhat shrunken, was washed with clear, hot water.

At this time I first thought of and saw what was left of the vermiform appendix, a little shriveled stump of which was all that remained. Fortunately, I did not know what to do with it and so did nothing but repack the cavity and continue treatment as before. The incidents calling for special treatment were unimportant. This patient had suffered some from diarrhea, but his bowels were kept quiet ten or twelve days after the operation and then moved without trouble or accident and recovery was satisfactory. I do not know why destruction processes had not occurred.

These cases demonstrate the fact that just a little knowledge is not always a very dangerous thing, and that water, cleanliness and drainage are important elements of success in surgical proceedings ; but they more definitely illustrate the difficulties that beset us in those days, by reason of the limited knowledge as to diagnosis and the scanty appliances at hand for operations and for subsequent treatment of our cases.

[NOTE.—This paper contained many observations of personal and local interest, but which, for lack of space, we are obliged to omit.—ED.]

